# Aboveground Net Primary Productivity in a Riparian Wetland Following Restoration of Hydrology

**DOI:** 10.3390/biology5010010

**Published:** 2016-02-04

**Authors:** Melissa Koontz, Christopher Lundberg, Robert Lane, John Day, Reza Pezeshki

**Affiliations:** 1 Department of Biological Sciences, The University of Memphis, Memphis, TN 38152, USA; pezeshki@memphis.edu; 2 Department of Oceanography and Coastal Sciences, Louisiana State University, Baton Rouge, LA 70803, USA; chrislundberg.phd@gmail.com (C.L.); rlane@lsu.edu (R.L.); johnday@lsu.edu (J.D.)

**Keywords:** wetland restoration, bottomland hardwood forest, wetlands, Mississippi River

## Abstract

This research presents the initial results of the effects of hydrological restoration on forested wetlands in the Mississippi alluvial plain near Memphis, Tennessee. Measurements were carried out in a secondary channel, the Loosahatchie Chute, in which rock dikes were constructed in the 1960s to keep most flow in the main navigation channel. In 2008–2009, the dikes were notched to allow more flow into the secondary channel. Study sites were established based on relative distance downstream of the notched dikes. Additionally, a reference site was established north of the Loosahatchie Chute where the dikes remained unnotched. We compared various components of vegetation composition and productivity at sites in the riparian wetlands for two years. *Salix nigra* had the highest Importance Value at every site. Species with minor Importance Values were *Celtis laevigata*, *Acer rubrum*, and *Plantanus occidentalis*. Productivity increased more following the introduction of river water in affected sites compared to the reference. Aboveground net primary productivity was highest at the reference site (2926 ± 458.1 g·m^−2^·year^−1^), the intact site; however, there were greater increase at the sites in the Loosahatchie Chute, where measurements ranged from 1197.7 ± 160.0 g m^−2^·year^−1^·to 2874.2 ± 794.0 g·m^−2^·year^−1^. The site furthest from the notching was the most affected. Pulsed inputs into these wetlands may enhance forested wetland productivity. Continued monitoring will quantify impacts of restored channel hydrology along the Mississippi River.

## 1. Introduction

The Mississippi River is the fourth longest and tenth largest river in the world. The Mississippi River Basin (MRB) is the fourth largest watershed in the world, draining more than 3,220,000 km^2^ covering about 40% of the landmass in the continental United States. The Lower Mississippi River sub-basin is characterized by low, flat topography, alluvial soils in a wide flood plain, relatively high rainfall, and high water tables [1]. The Mississippi Alluvial Valley (MAV) once contained nearly 10 million ha of bottomland hardwood forests, representing the largest area of bottomland hardwood wetlands in North America [2]. Historically, black willow stands in the rive floodplain occurred in low moist spots in parts of the alluvial valley near river channels [3]. Settlement along the Mississippi River led to intensive harvesting of forests, isolation of much of the original floodplain with levees, the development of large-scale agricultural practices, urbanization, and the draining of wetlands [4]. The Mississippi River and Tributaries Project included the levee system and many stone dikes in order to divert most river flow from secondary channel complexes into the main navigation channel [1]. Dams and reservoirs, especially in the Missouri basin, resulted in the retention of sediment stored in pools behind the dams and in the fields of the winged dams, which enhances the flow magnitude in the main channel [5]. This has resulted in many of the secondary channels drying completely during the year, resulting in degraded riparian habitat including the loss of aquatic habitat [1]. Prior to settlement, these bottomland hardwood forests stored 60 days of river discharge compared to 12 days following the leveeing of the river [6].

By the 1980s, an estimated 2.8 million ha of bottomland hardwood forest remained of the original 10 million ha, and much of the habitat that has remained is highly fragmented and hydrologically altered [2,7,8]. Approximately 58% of the MRB is now utilized as cropland, with 90 to 95% of land use in many subwatersheds being dominated by corn (*Zea mays* L.) and soybeans (*Glycine max* (L.) Merr.) [9]. Effectively, the large contiguous forested wetland ecosystem was converted into a system of fragmented forested wetlands embedded within a larger agricultural matrix [2]. Agriculture, both landscape alteration and practices, has had a broad range of negative impacts on ecosystem processes in wetlands [10]. The shift of land use from wetlands to agricultural systems has led to a reduction of ecosystem goods and services, such as wildlife habitat, carbon sequestration, sediment retention, nutrient cycling, flood regulation, and water treatment [11,12].

The major driver of degradation for the Gulf of Mexico is upstream water management and agricultural practices in the MRB [13]. As land drainage has increased for agriculture, so has nitrogen (N) application, especially nitrate (NO_3_^−^), and subsequent transport of N via the Mississippi River to the Gulf of Mexico causing hypoxia in the nearshore waters off Louisiana [14,15,16]. Although hypoxia in the northern Gulf of Mexico is a result of a number of interacting factors [17], such as the development of a pycnocline, nutrient input from the MRB is a major driver [16]. Watersheds in the Lower and Central Mississippi and Ohio River basins were ranked the highest contributors of total N and total phosphorus (P) being transported to the Gulf of Mexico [18]. The high correlation between May-June nutrient load and May–June streamflow, most likely due to high freshwater runoff mobilizing nutrients in the watershed [17]. These conditions create a hypoxic zone along the coast that has grown to as high as 20,000 km^2^ during midsummer, currently the second largest in the world in area [16,19,20,21,22].

Wetlands provide a vital ecosystem service of improving water quality by treating and removing a variety of waste products with reported reductions of NO_3_^−^ by more than 80% [10]. Undisturbed wetlands experience flood pulsing that facilitates an exchange of materials between rivers and their floodplain [23,24,25], providing mineral sediments and nutrients necessary for plant growth [26,27]. The creation and restoration of wetlands in the Mississippi-Ohio-River basin has been recommended to intercept and reduce excess N concentrations in the Mississippi River [28,29,30]. Hypoxia in the Gulf of Mexico could be significantly reduced with an estimated 2 million ha of wetlands strategically placed in the agricultural watershed [29]. Wetland ecosystems provide services that benefit human welfare by mediating energy and material flow such as water quality improvement due to reduction of suspended solids, biochemical oxygen demand, N, P, and heavy metals, and other pollutants [1,31,32]. Mature bottomland hardwood forest stands foster snag and cavity development, increased inputs of woody debris and leaf litter, increased vertical and horizontal structure, and increased diversity of shrubs and understory plants [2].

Conceptually, our understanding river ecosystems includes both the River Continuum Concept (RCC, [33]) where the longitudinal pattern changes from upstream to downstream are emphasized to the Flood Pulse Concept (FPC, [23,34,35]) where horizontal exchange between a river and its floodplain is emphasized as the main factor determining the function of both the river and its adjacent riparian floodplains. Changes in the river system have altered both upstream-downstream and river-floodplain interactions.

**Figure 1:**
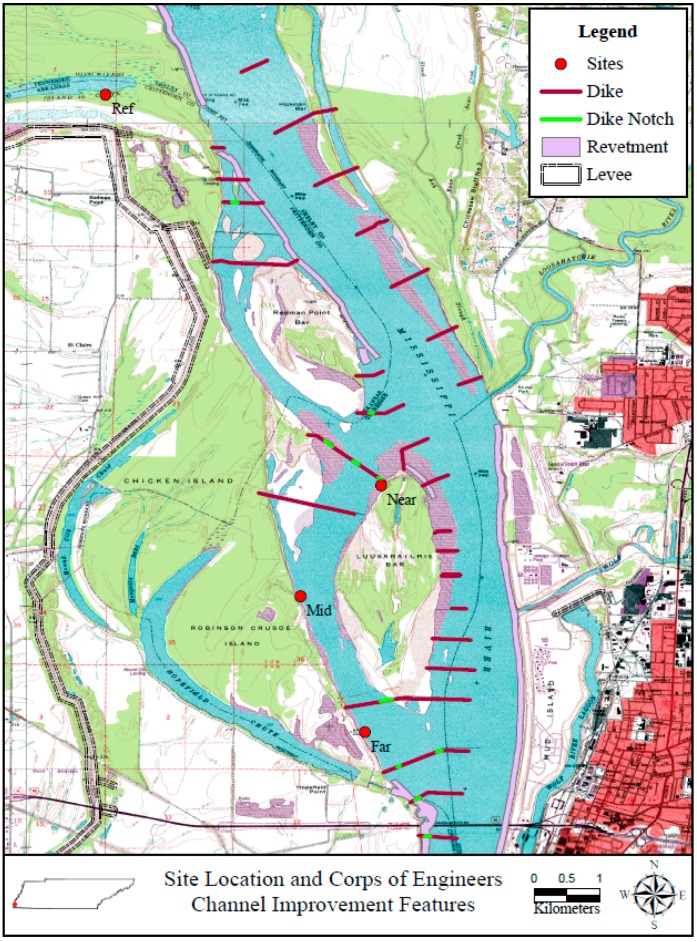
Map of site locations in relation to notched dikes on the Mississippi River. Sites are represented by red dots, and notches are green dashes.

The main purpose of the notching project was to restore flow for the benefit of two federally listed species, the Pallid Sturgeon (*Scaphirhynchus albus*) and the Interior Least Tern (*Sterna antillarum athalassos*), as well as other recreationally and commercially valuable wildlife. In the fall of 2008, twelve notches were excavated in nine existing dikes at Loosahatchie Chute to restore flow to more than 17 km of secondary channel (Figure 1). The objective of this study was to measure the impact of notching the rock dikes on the forested wetlands located along the margins of the Loosahatchie Chute and determine if the reintroduction of flood pulses would impact species composition and the changes in plant production. This study was part of a larger study that involved measurement of nutrient dynamics, wetland productivity, and greenhouse gas emissions at three sites in the MRB [36]. The specific objective was to determine spatial and temporal patterns of productivity and structure of the forest in response to restored hydrology. We hypothesized that aboveground net primary productivity would increase at the restored sites. Increased productivity would indicate that the restored hydrology was positively impacting wetland function.

## 2. Methods

### 2.1. Study Area

**Figure 2:**
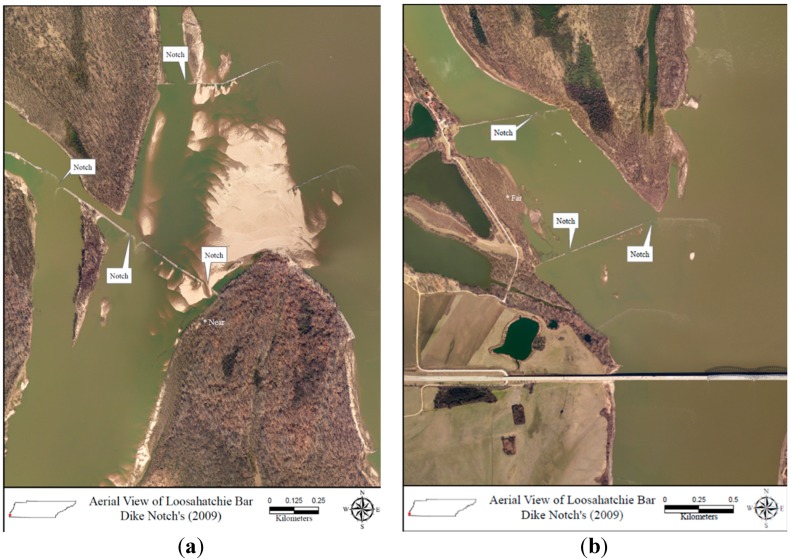
Aerial view of dikes notched near the (**a**) north end and (**b**) south end of the Loosahatchie Chute following restored flow to secondary channel complexes along the Mississippi River. Bridge in (**b**) is Interstate 40.

Loosahatchie Bar is located on the west bank of the Mississippi River, opposite Memphis, Tennessee, between river miles 736.5 and 742.8 on the border of Shelby County, Tennessee, and Crittenden County, Arkansas (Figure 1, note: river miles are the official designation of distance along the Mississippi and are used here so that the location of our study can easily be located in the context of the larger system). Stone dikes were constructed during the 1960s by the United States Army Corps of Engineers (USACE) to keep water from the Mississippi River from flowing through the Redman Point Loosahatchie Bar secondary channel complex (herein referred to as the Loosahatchie Chute). In an attempt to restore the area, twelve notches were excavated by the USACE from nine of the existing dikes at Loosahatchie Chute during October 2008–February 2009 (Figure 1). Dikes measure between 100 and 600 m in length, 7.5 to 60 m wide at top, and 20 to 64 m wide at the bottom. Each notch was 0.9 to 3.3 m deep. These notches restored flow in more than 17 km of secondary channel in the Loosahatchie Chute (Figure 2). This effort was funded by the US Fish and Wildlife Service Fish Passage Program, the Audubon Society, and non-governmental conservation organizations in order to restore flow to habitats of two federally listed species (*S. albus* and *S. antillarum athalassos*), as well as other wildlife.

### 2.2. Site Description

**Figure 3:**
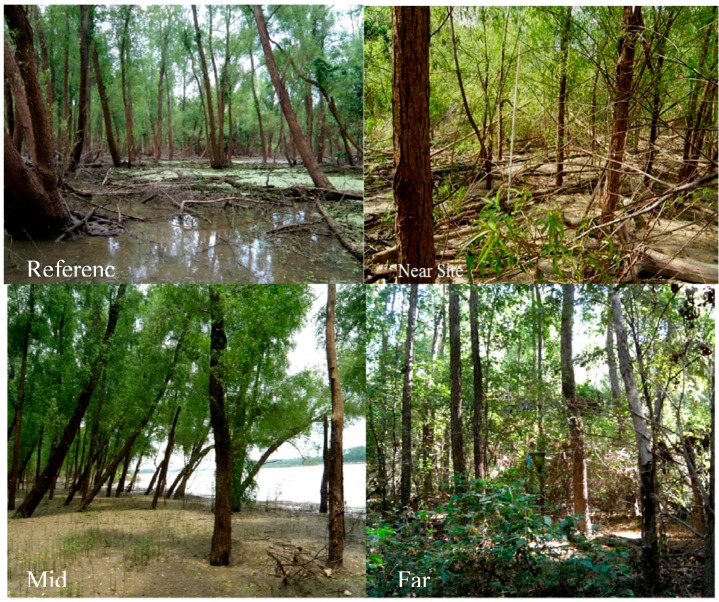
Images of each of the sampling locations relative to notched winged dams near the Loosahatchie Bar on the Mississippi River.

Study site selection and data collection began February 2009. Sites were selected in relation to the Redman Point-Loosahatchie Bar Environmental Restoration Project (Figure 1; Table 1). Three study sites (Near, Mid and Far) were established along the Loosahatchie Chute, and a reference site (Ref) was positioned north of the Redman point dikes. All sites were riparian forest composed primarily of black willow (*Salix nigra* Marsh.; Figure 3). Three 25 × 25 m plots were established at each site for measurement of net primary productivity. The number and size of the plots were established to quantify biomass change as a representation of the area being measured. It is important to note that logistics and access constrained the selection of sites. During high discharge of the river, high river stages and strong current limit access to many areas due to safety concerns. By contrast, during low flow, boat access is limited due to low water and it was practically impossible to go to many areas on foot. For these reasons, the reference site was less directly impacted by river flow than the study sites.

**Figure 4:**
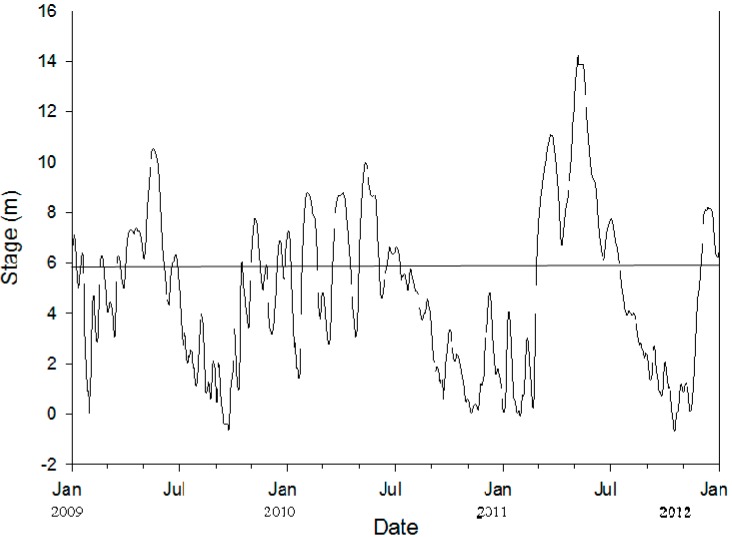
Mississippi River stage at Memphis, Tennessee, measured at the Weather Bureau Gage, where the National Weather Stage Gage height of 0 m is measured at an elevation of 56.1 m; this is based on older United States Geological Survey (USGS) topographic maps and NGVD29 benchmarks. Flood stage is at a gage height of 10.4 m. This is river stage data from late January 2009 through January 2012. Sites are inundated at approximately 5.5 m, indicated by the solid line.

The river stage for the Mississippi River at Memphis, Tennessee, was measured at the Weather Bureau Gage, where the National Weather Stage Gage height of 0 m is measured at an elevation of 56.1 m; this is based on older USGS topographic maps and NGVD29 benchmarks. Flood stage is at gage height 10.4 m. Initial measurements began during winter 2009 and continued until autumn 2011 (Figure 4). Sites were usually inundated at river gage height of approximately 5.5 m. All sites experienced intermittent flooding. The river stage in 2009 ranged from −0.65 to 10.55 m and averaged 4.67 ± 0.13 m, with the sites flooded 154 days of the year, or 42% of the time. In 2010 the river stage ranged from −0.05 to 9.98 m and averaged 4.61 ± 0.14 m, with the sites flooded 37% of the time (135 days a year). In 2011 the river stage ranged from −0.70 to 14.27 m and averaged 5.28 ± 0.21 m with flooding of the sites 48% of the time (175 days a year).

**Table 1:** Coordinates and elevations of study field sites.

Site	Latitude	Longitude	Elevation (m)
Ref	35°15′14.26″ N	90°06′51.23″ W	62.64
Near	35°12′00.04″ N	90°04′34.55″ W	61.75
Mid	35°11′05.04″ N	90°05′14.56″ W	61.84
Far	35°09′57.44″ N	90°04′42.58″ W	62.94

### 2.3. Soil

Soil was classified according to the United States Department of Agriculture (USDA) National Resources Conservation Service Web Soil Survey. The Ref site soil data were collected 17 November 2008. The soil was classified as Bowdre silty clay and was frequently flooded. At a depth from 0 to 12.7 cm, the cation-exchange capacity (CEC) was 25 to 45 meq 100 g^−1^ with a pH of 5.6 to 7.3; at a depth from 12.7 to 43.2 cm, the CEC was 22–40 meq 100 g^−1^ with a pH of 5.6 to 7.3; at a depth from 43.2 to 106.7 cm, the CEC was 7.0 to 18 meq 100 g^−1^ with a pH of 6.1 to 8.4; and at a depth from 106.7 to 152.4 cm, the CEC is 5.0 to 18 meq 100 g^−1^ with a pH of 6.1 to 8.4. The soil at the Mid site was classified as Crevasse fine sand from data collected 23 September 2008. At a depth from 0 to 20.3 cm, the CEC was 1.8 to 6.8 meq 100 g^−1^ with a pH of 5.6 to 8.4; and at a depth from 20.3 to 152.4 cm, the CEC was 1.4 to 6.1 meq 100 g^−1^ with a pH of 5.6–8.4. Soil at the Near and Far sites have not been surveyed.

### 2.4. Aboveground Productivity

**Figure 5:**
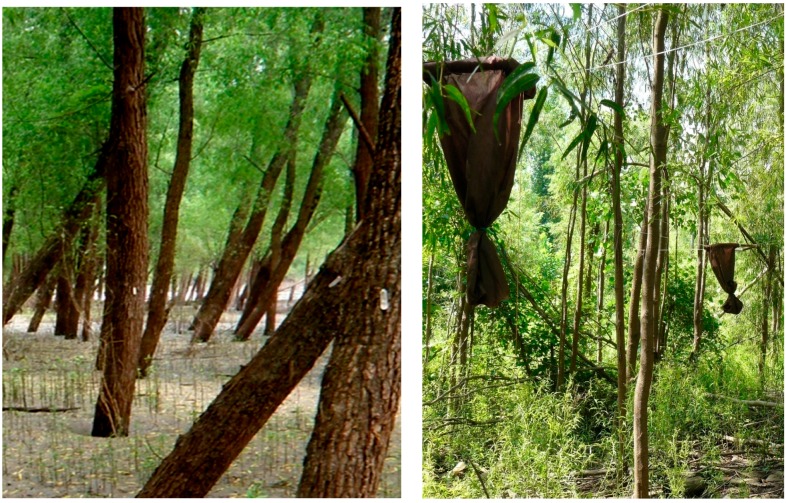
Tagged trees (**left**) and leaf litter collection traps (**right**).

Total aboveground NPP was calculated as the sum of stem growth and litterfall. Woody stem growth productivity was calculated using diameter at breast height (dbh) measurements of all trees with dbh greater than 10.0 cm. Trees were tagged with an aluminum identification tag positioned ~1.3 m from the base of the tree (Figure 5, left; [37,38,39]). Forest composition including species composition, percent cover, basal area and importance values were quantified at each study site as described below. Dbh was measured above and below (~5 cm) the nail holding the identification tag [40]. Diameter tape (d-tape) designed to read dbh from circumference was used to take measurements. Measurements of dbh began in the winter of 2009 and were taken yearly in the winters of 2010 and 2011. Woody biomass was calculated using species—specific allometric equations based on stem dbh (average of top and bottom measurement) as the independent variable (Table 2; [40,41]. Biomass was summed at each of the three plots at each study site and divided by the area of the plot (625 m^2^) resulting in the biomass per unit area (kg·m^−2^) for each plots for both years.

**Table 2:** Regression equations used to convert diameter at breast height (DBH) measurements of trees to whole tree biomass.

Species	y = f(DBH)	DBH Range	Reference
*Salix nigra*	Biomass(kg) = 10^((−1.5 + 2.78 × LOG10(DBHcm)))	n.a.	[41]
*Acer rubrum*	Biomass (kg) = ((2.39959 × ((DBHcm × 0.394)^2)^1.2003)) × 0.454	10–28 cm	[40]
Other Species	Biomass (kg) = ((2.54671 × ((DBHcm × 0.394)^2)^1.20138)) × 0.45	10–28 cm	[40]

Litterfall productivity was measured using five leaf litter collection traps placed randomly within each plot, totaling 15 traps per site (5 per subplot). The collection traps were constructed from 1.91 cm diameter polyvinyl chloride (PVC) pipe, measuring 0.25 × 0.25 m, with a screened net bottom. Each trap was elevated (~3 m) above the ground to prevent inundation during high water periods (Figure 5, right). The traps were installed in June 2009. During the high litterfall period from October to December, litter was collected approximately every two weeks until all leaves had dropped from the trees. Litter was not collected during the rest of the year due to spring floods washing away most of the traps and inaccessibility of the sites. Leaf litter was separated from woody litter and dried to constant mass at 65 °C. Final dry weights were weighed to the nearest 0.01 g.

Tree species composition analysis used equations 1–3 modified from Barbour *et al.* (1987) [42]. Basal area was defined as the trunk cross-sectional area of a given species (*i.e.*, cm^2^·m^−2^). Importance Values equal the sum of relative density and relative dominance for a maximum value of 2.

**Relative density** = (individuals of a species)/(total individuals of all species) (1)

**Relative dominance** = (total basal area of a species)/(total basal area of all species) (2)

**Importance** = Relative density + Relative dominance (3)

### 2.5. Data Analyses

To determine if restoring hydrology had increased productivity, NPP among sites within each sampling year was tested with general linear model for analysis of variance (ANOVA; [43]). Significant results were followed by Tukey’s post-hoc analysis [44]. NPP for both 2010 and 2011 were compared at each site using paired sample *t*-test. Differences were considered significant at α < 0.05.

## 3. Results

Trees at the Near site were more exposed to wind compared to the other sites, which were protected by surrounding forests, contributing to stem breaks and canopy debris on the forest floor. Tree density at all sites was dominated by *S. nigra*, which also had the highest Importance Value for every site (Table 3). Species with minor Importance Values identified at some sites included sugarberry (*Celtis laevigata* Willd.), red maple (*Acer rubrum* L.), American elm tree (*Ulmus americana* L.), and sycamore (*Platanus occidentalis* L.)

The Ref site had the lowest tree density of 296 trees ha^−1^, however, these were the largest trees increasing in basal area from 3.26 cm^2^·m^−2^ in 2009 to 3.47 cm^2^·m^−2^ in 2010 and 3.67 cm^2^·m^−2^ in 2011 (Table 3). The relative density and relative dominance of *S. nigra* was 100% for 2009–2011, with an importance value of 2 for each year (Table 3).

Table 3Tree species, tree density, basal area, relative density, relative dominance, and importance values results from each study site.

**Table:** 

Site	Species	Tree Density (Trees ha^−1^)			Basal Area (cm^2^·m^−2^)		
		2009	2010	2011	2009	2010	2011
Ref	*Salix nigra*	298	298	293	3.26	3.47	3.67
Near	*Salix nigra*	533	528	496	0.63	0.76	0.86
Near	*Celtis laevigata*	10	10	10	0.01	0.02	0.02
Mid	*Salix nigra*	314	314	314	2.84	3.02	3.22
Mid	*Platanus occidentalis*	5	5	5	0.01	0.01	0.01
Far	*Salix nigra*	352	352	352	2.39	2.55	2.78
Far	*Acer rubrum*	64	64	58	0.10	0.11	0.12
Far	*Ulmus americana*	5	5	5	0.02	0.02	0.03

**Table:** 

Site	Species	Relative Density			Relative Dominance			Importance Value		
		2009	2010	2011	2009	2010	2011	2009	2010	2011
Ref	*Salix nigra*	100%	100%	100%	100%	100%	100%	2	2	2
Near	*Salix nigra*	98%	98%	98%	98.4%	97.4%	97.7%	1.96	1.95	1.96
Near	*Celtis laevigata*	2%	2%	2%	1.6%	2.6%	2.3%	0.04	0.05	0.04
Mid	*Salix nigra*	98.3%	98.3%	98.3%	99.6%	99.7%	99.7%	1.98	1.98	1.98
Mid	*Platanus occidentalis*	1.7%	1.7%	1.7%	0.4%	0.3%	0.3%	0.02	0.02	0.02
Far	*Salix nigra*	83.5%	83.5%	84.6%	95.2%	95.2%	94.9%	1.79	1.79	1.80
Far	*Acer rubrum*	15.2%	15.2%	14.1%	4%	4.1%	4.1%	0.19	0.19	0.18
Far	*Ulmus americana*	1.3%	1.3%	1.3%	0.8%	0.7%	1%	0.02	0.02	0.02

The Near site had nearly twice the density of trees than the Ref site and approximately a quarter of the basal area of trees growing at the other sites (Table 3). There was a decline in tree density at the Near site from 543 trees ha^−1^ in 2009 to 506 trees ha^−1^ in 2011. This particular site experiences direct winds from the west, which are strong enough to snap tree stems. Saplings were observed in the understory, which will grow to occupy the spaces opened from the damaged trees, however, the DBH measurements of these trees had not yet reached 10 cm. The basal area of *S. nigra* at the Near site increased from 0.63 cm^2^·m^−2^ in 2009 to 0.76 cm^2^·m^−2^ in 2010 and 0.86 cm^2^·m^−2^ in 2011 (Table 3). The basal area of *C. laevigata* in 2009 was 0.01 cm^2^·m^−2^ and increased slightly up to 0.02 cm^2^·m^−2^ in 2010 and 2011. This decrease in density and basal area at the Near site was reflected in the stem growth measurements. The relative density of *S. nigra* was 98% and *C. laevigata* was 2% for 2009–2011 (Table 3). The relative dominance of *S. nigra* was approximately 98% and *C. laevigata* was approximately 2% for all three years (Table 3). At the Near site *S. nigra* was the most important species (1.96) with *C. laevigata* being of minor importance (0.04) for 2009–2011 (Table 3).

The Mid site maintained a density of 319 trees ha^−1^ during all three years (Table 3). The basal area of *S. nigra* at the Mid site increased from 2.84 cm^2^·m^−2^ in 2009 to 3.02 cm^2^·m^−2^ in 2010 and 3.22 cm^2^·m^−2^ in 2011 (Table 3). The basal area of *P. occidentalis* in 2009 was 0.01 cm^2^·m^−2^ for each year. The relative density of *S. nigra* was 98.3% and *P. occidentalis* 1.7% for 2009–2011 (Table 3). The relative dominance of *S. nigra* was approximately 99.7% and *P. occidentalis* was approximately 0.3% for all three years (Table 3). At the Mid site *S. nigra* was the most important species (1.98) with *P. occidentalis* being of minor importance (0.02) for 2009–2011 (Table 3).

The Far site averaged 419 trees ha^−1^. *S. nigra* increased in basal area from 2.39 cm^2^·m^−2^ in 2009 to 2.78 cm^2^·m^−2^ in 2011 (Table 3). The basal area increased for *A. rubrum* from 0.10 cm^2^·m^−2^ to 0.12 cm^2^·m^−2^ and for *U. americana* from 0.02 cm^2^·m^−2^ to 0.03 cm^2^·m^−2^ from 2009 to 2011. The relative density ranges were 83.5%–84.6% for *S. nigra*, 14.1–15.2% for *A. rubrum*, and 1.3% for *U. americana* for 2009–2011 (Table 3). The relative dominance of *S. nigra* ranged from 94.9%–95.2%, *A. rubrum* ranged from 4.0%–4.1%, and *U. americana* ranged from 0.7%–1.0% for all three years (Table 3). At the Far site *S. nigra* was the most important species (1.79–1.80) with *A. rubrum* (0.18–0.19) and *U. americana* (0.02) being of minor importance for 2009–2011 (Table 3).

**Figure 6:**
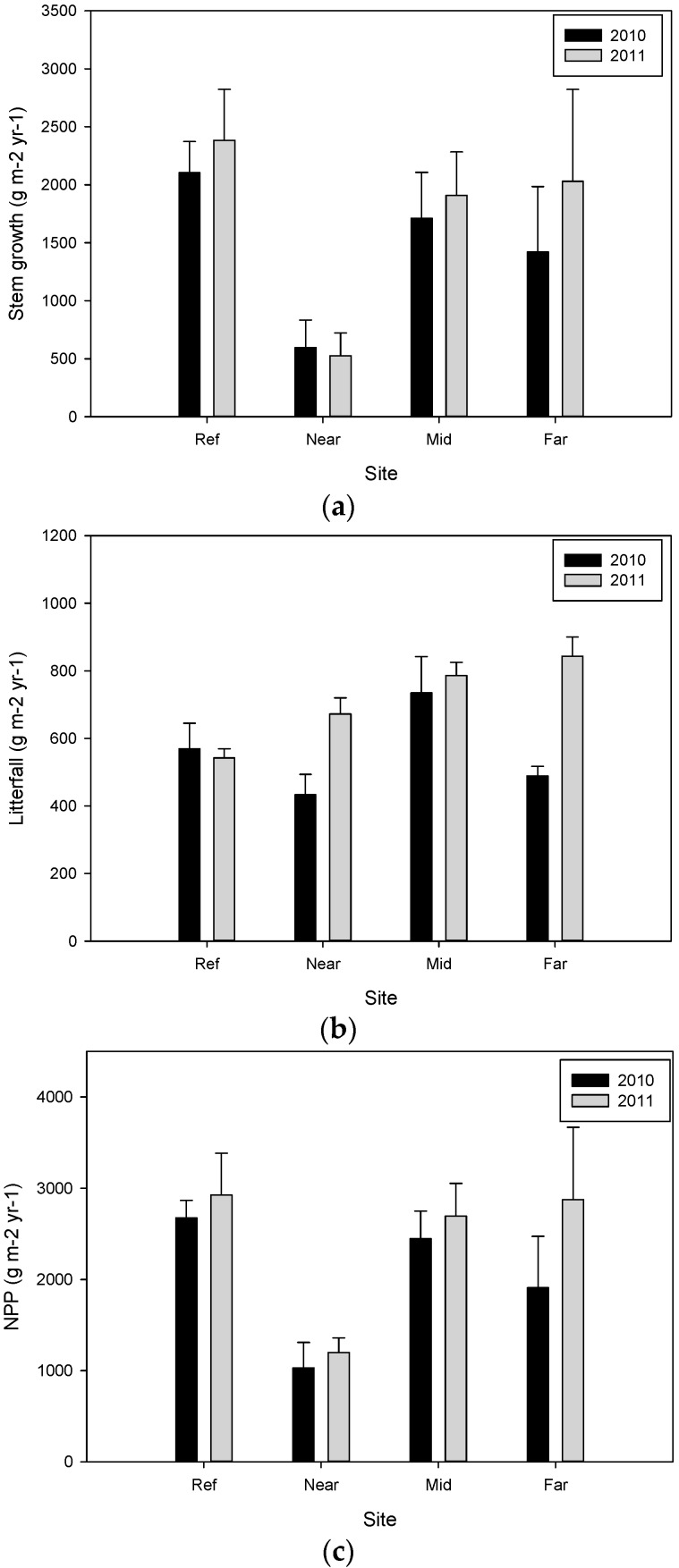
Comparison of (**a**) stem growth; (**b**) litterfall; and (**c**) total aboveground net primary productivity (NPP) measured in g m^−2^·year^−1^ per plot for restored forested wetlands along the Mississippi River in the beginning of 2010 and 2011. Each value is the mean of the measurements (±se). Significant differences between years were not detected, according to paired sample *t*-test. Differences were considered significant at α < 0.05.

Stem growth in 2010 ranged from 595.2 ± 237.6 g m^−2^·year^−1^ at the Near site to 2105.1 ± 267.1 g m^−2^·year^−1^ at the Ref site, however, differences were not significant between sites (F_3,8_ = 2.714, *p* = 0.115; Table 4; Figure 6a). In 2011 stem growth ranged from 525.5 ± 195.8 g m^−2^·year^−1^ at the Near site to 2383.4 ± 439.6 g m^−2^·year^−1^ at the Ref site, and again there was not a significant difference between sites (F_3,8_ = 2.667, *p* = 0.119). For all sites combined, there was no significant difference in stem growth increase in 2011 compared to 2010 (t_11_ = −1.446, *p* = 0.176).

Litterfall collected in 2010 ranged from 433.0 ± 60.5 g m^−2^·year^−1^ at the Near site to 734.5 ± 107.3 g·m^−2^·year^−1^ at the Mid site, and was not significantly different between sites (F_3,8_ = 3.180, *p* = 0.085; Table 4). In 2011 litterfall was different between sites (F_3,8_ = 9.092, *p* = 0.006), with both the Far (843.1 ± 57.0 g m^−2^·year^−1^; *p* = 0.006) and the Mid sites (786.0 ± 39.3 g m^−2^·year^−1^; *p* = 0.019) being significantly more productive than the Ref site (542.7 ± 26.5 g m^−2^·year^−1^). The Near site (672.2 ± 47.6 g·m^−2^·year^−1^) was not significantly different from the other sites. For all sites combined, litterfall increased in 2011 compared to 2010 (t_11_ = −2.921, *p* = 0.014). The largest change in litterfall was the increased productivity at the Far site (t_2_ = −12.539, *p* = 0.006) followed by the Near site (t_2_ = −5.039, *p* = 0.037; Figure 6b). There was no change in litterfall at the Mid site (t_2_ = −0.600, *p* = 0.610) or the Ref site (t_2_ = −0.346, *p* = 0.762).

Total aboveground NPP differed between sites during the first year (F_3,8_ = 4.064, *p* = 0.050; Table 4). NPP was highest at the Ref site in 2010 (2674.4 ± 192.6 g m^−2^·year^−1^) and lowest at the Near site (1028.2 ± 280.8 g m^−2^·year^−1^; *p* = 0.05). Both the Mid (2446.1 ± 303.1 g m^−2^·year^−1^) and Far (1907.7 ± 564.8 g m^−2^·year^−1^) sites did not differ from each other or the other sites in NPP. In 2011 aboveground NPP ranged from 1197.7 ± 160.0 to 2926.1 ± 458.1 g m^−2^·year^−1^, however, there was no significant difference between sites (F_3,8_ = 2.726, *p* = 0.114; Table 4).

**Table 4:** Summary of average woody stem growth, litterfall, and total aboveground net primary productivity (NPP) per plot for restored forested wetlands along the Mississippi River. Each value is the mean for the measurements (±se). Sites with significant differences are identified by lowercase letters, a or b. This is calculated according to Tukey’s post-hoc analysis. Differences were considered significant at α < 0.05.

Site	Plot Number	Year 2009–2010 (g·m^−2^·year^−1^)			Year 2010–2011 (g·m^−2^·year^−1^)		
		Stem Growth	Litterfall	NPP	Stem Growth	Litterfall	NPP
Ref	3	2105.1 ± 267.1	569.3 ± 75.4	2674.4 ± 192.6 ^a^	2383.4 ± 439.6	542.7 ± 26.5 ^b^	2926.1 ± 458.1
Near	3	595.2 ± 237.6	433.0 ± 60.5	1028.2 ± 280.8 ^b^	525.5 ± 195.8	672.2 ± 47.6 ^a,b^	1197.7 ± 160.0
Mid	3	1711.6 ± 395.8	734.5 ± 107.3	2446.1 ± 303.1 ^a,b^	1909.3 ± 374.6	786.0 ± 39.3 ^a^	2695.3 ± 357.9
Far	3	1419.2 ± 565.0	488.5 ± 29.0	1907.7 ± 564.8 ^a,b^	2031.1 ± 792.2	843.1 ± 57.0 ^a^	2874.2 ± 794.0

NPP increased in 2011 compared to 2010 (t_11_ = −2.257, *p* = 0.05; Figure 6c). The Near site had a 45% increase (239.2 g m^−2^·year^−1^) in litterfall, contributing 56% of total NPP, compared to 42% contribution the previous year. Total NPP increased at the Near site by 169.5 g m^−2^·year^−1^ despite the loss of 69.70 g m^−2^·year^−1^ in stem productivity. The Mid site had increased production in both litterfall (51.5 g m^−2^·year^−1^) and stem growth (197.7 g m^−2^·year^−1^), and thus in total NPP (249.2 g m^−2^·year^−1^). The Far site had the greatest changes in both litterfall (354.6 g m^−2^·year^−1^) and stem growth (611.9 g m^−2^·year^−1^), which is reflected in the 50% increase in the total NPP (966.5 g m^−2^·year^−1^). The first year the Far site had 766.7 g m^−2^·year^−1^ less total NPP than the Ref site, and following hydrologic restoration it was within 51.9 g m^−2^·year^−1^ of total NPP compared to the Ref site. Total NPP at the Ref site increased the least (11.4%, 251.70 g m^−2^·year^−1^), having an increase in stem growth of 278.3 g m^−2^·year^−1^ and a decrease in litterfall of 26.6 g m^−2^·year^−1^.

## 4. Discussion

The impact of the reintroduction of flood pulses to the functioning of the bottomland hardwood forests measured by species composition and the changes in plant production rates was detectable. The dominance of *S. nigra* at all sites in the present study reflects past plant community composition of similar locations at the same elevation and river stage. Historically, the Mississippi River floodplain has had black willow stands in low moist spots in parts of the alluvial valley near river channels, where water levels are 4.6 to 9.1 m above mean low water [3].

The frequency and duration of water availability to the Loosahatchie Chute increased due to notching and this was reflected by the response of tree productivity. Initially, measures of NPP indicated there were differences between the sites, with the greatest difference being between the Ref and Near sites. Following hydrologic restoration, the NPP was not significantly different between sites due to the increased productivity of all the restored sites. The restored study sites generally had a greater percentage increase compared to the reference site. This finding of increased productivity is important. Even though the Ref site had the greatest overall NPP for both years, it had the least amount of change. A previous study of Mississippi delta freshwater forested wetlands, which included *S. nigra*, receiving municipal effluent discharge had a measured reduction of an average of 79% of total Kjeldahl nitrogen and an average of 88.5% total phosphorus, where sites nearest the secondarily treated municipal effluent had increased productivity of vegetation compared to control sites [45]. In that study, the site closest to receiving the discharge had greater litterfall (717 g·m^−2^·year^−1^) and total NPP (1467 g m^−2^·year^−1^) than a control site (412 and 714 g m^−2^·year^−1^, respectively), and stem growth at all sites ranged between (302–776 g m^−2^·year^−1^; [45]). In another previous study of seasonally flooded forests in Louisiana, bottomland hardwood woody productivity averaged 574 g·m^−2^·year^−1^ litterfall, 800 g·m^−2^·year^−1^ stem growth, and 1374 g·m^−2^·year^−1^ total NPP [46]. The present study had similar measurements in productivity as the forested wetlands in the delta region (Table 4). In general, it has been found that the most productive forested wetland sites are those with intermediate flooding [6,46]. In general, the values for total NPP from this study are in the upper range of those reported in the literature [6,46]. This is likely related to the seasonal flooding as well as nutrient supply from the river.

Measurements of the soil structure at the sites revealed that the restored sites had a higher proportion of silt than sand and clay particles [47]. The restored sites, beginning with the Near site, had greater reductions of total carbon, nitrogen, and phosphorus with decreased reduction down the channel [47]. The Ref site had a higher percentage of clay than other soil particles, and higher concentrations of all the nutrients measured [47]. Floodplain fertility depends on quality of deposited sediment and dissolved inorganic compounds, including plant nutrients, being replenished and deposited by inflowing river water [23].

The effectiveness of functions of restored hydrology that maintains restored wetland flora and soil carbon resources may take centuries to return to a healthy state [48]. As ecosystems age, they tend to grow in increasing orders of complexity. The pulsing of water and nutrients during seasonal flooding, followed by the released nutrients and dry period, promote the enhanced productivity of seasonally flooded wetlands [6]. Swamps with flowing water and seasonal flooding regimes have higher forest productivity [46]. Continued study of net primary productivity and tree communities at these sites will help quantify impacts of the restored flood pulse over time following restored channel hydrology. It is possible that a long-term evaluation of tree growth at these sites would indicate differences among sites. Restored hydrology to secondary channels can improve habitat for federally endangered and commercially important species, provide public benefits include boating, fishing, and birding, and improve water quality through wetland filtration. Flood pulses are an important, integral part of the Mississippi River floodplain, having a hydrological effect on the biota, improving system functioning [23]. These initial results indicate that restoration via notching of weirs is promising.

The functioning of the LC and the effectiveness of the restoration can be understood within the context of the River Continuum Concept (RCC; [33]) and the Flood Pulse Concept (FPC; [23,34,35]. In the RCC, longitudinal pattern changes from upstream to downstream are emphasized. Construction of rock weirs led to a reduction in upstream-downstream connectivity. This has impacted the productivity of the floodplain forest ecosystem as well as leading to negative impacts on the Pallid Sturgeon and the Interior Least Tern. Our results suggest that restoring the connectivity of the river by notching rock weirs led to greater increases in productivity as a result of enhanced exchange between the river and its floodplain as described by the FPC. Future measurements of the area will allow the determination of long-terms benefits of this river reconnection project.

## 5. Couclusions

The initial results of the effects of hydrological restoration on forested wetlands in the Mississippi alluvial plain near Memphis, Tennessee showed that enhancing the connectivity of the river by notching rock weirs led to greater increases in productivity of the study sites as a result of improved exchange between the river and its floodplain. The site furthest from the notching was the most affected. Pulsed inputs into these wetlands appeared to have enhanced forested wetland net primary productivity. However, continued monitoring is needed to quantify the long-term impacts of restored channel hydrology on forest productivity.
